# Skin Imaging Using Ultrasound Imaging, Optical Coherence Tomography, Confocal Microscopy, and Two-Photon Microscopy in Cutaneous Oncology

**DOI:** 10.3389/fmed.2019.00274

**Published:** 2019-11-22

**Authors:** Byung Ho Oh, Ki Hean Kim, Kee Yang Chung

**Affiliations:** ^1^Department of Dermatology and Cutaneous Biology Research Institute, Yonsei University College of Medicine, Seoul, South Korea; ^2^Department of Mechanical Engineering, Pohang University of Science and Technology, Pohang-si, South Korea

**Keywords:** skin imaging, skin cancer, benign skin tumor, ultrasound, optical coherence tomography, confocal microscopy, two-photon microscopy

## Abstract

With the recognition of dermoscopy as a new medical technology and its available fee assessment in Korea comes an increased interest in imaging-based dermatological diagnosis. For the dermatologist, who treats benign tumors and malignant skin cancers, imaging-based evaluations can assist with determining the surgical method and future follow-up plans. The identification of the tumor's location and the existence of blood vessels can guide safe treatment and enable the use of minimal incisions. The recent development of high-resolution microscopy based on laser reflection has enabled observation of the skin at the cellular level. Despite the limitation of a shallow imaging depth, non-invasive light-based histopathologic examinations are being investigated as a rapid and pain-free process that would be appreciated by patients and feature reduced time from consultation to treatment. In the United States, the current procedural terminology billing code was established for reflectance confocal microscopy in 2016 and has been used for the skin cancer diagnosis ever since. In this review, we introduce the basic concepts and images of ultrasound imaging, optical coherence tomography, confocal microscopy, and two-photon microscopy and discuss how they can be utilized in the field of dermatological oncology.

## Introduction

Efforts to diagnose skin cancer without skin biopsy are ongoing. The diagnoses of patients with suspected skin cancer are confirmed by punch biopsy followed by histopathological examination, which involve the collection of a small portion of the entire lesion to diagnose skin cancer (1). In this case, since only vertical information of a specific region is acquired, dermoscopy can supplement horizontal information of the entire lesion to identify the most suitable biopsy site. However, dermoscopy has an inherent depth limit confined to the upper dermis (Table 1).

**Table 1:** Pros and cons of skin biopsy and dermoscopy.

	**Skin biopsy**	**Dermoscopy**
Advantages	1. Provide universal validity based on long-term accumulated histopathological criteria	1. Identify optimal biopsy sites 2. Reduce unnecessary biopsy 3. Determine horizontal extent of skin lesion 4. Continue to observe lesion treatment
Disadvantages	1. Limitation of evaluating whole lesion by vertical information of specific region 2. Limitations of repeated practice due to pain, bleeding, and infection risk	1. Inherent depth limitation (upper dermis) 2. Difficulty implementing 3D image 3. No reflection of functional and dynamic information (blood flow velocity, oxygen saturation, etc.) of the skin

To observe lesions deep to the upper dermis, the maximum depth that can be observed with dermoscopy, non-invasive techniques, such as confocal microscopy, multiphoton microscopy, optical coherence tomography, and ultrasound must be used. Although each operation principle is different, they all use the reflection characteristic as if it is mirrored, and the skin's depth and resolution differ among device types (Table 2). Here we briefly discuss each available device and its clinical use in the dermatology field.

**Table 2:** Device resolution and imaging depths^1^.

	**Resolution**	**Penetration depth**
Confocal microscopy	1 μm	~500 μm
Optical coherence tomography	2–10 μm	~2 mm
Ultrasonography	150 μm	~10 cm
High-resolution computed tomography	300 μm	Entire body
Magnetic resonance imaging	1 mm	Entire body

## Ultrasound Imaging

**Figure 1:**
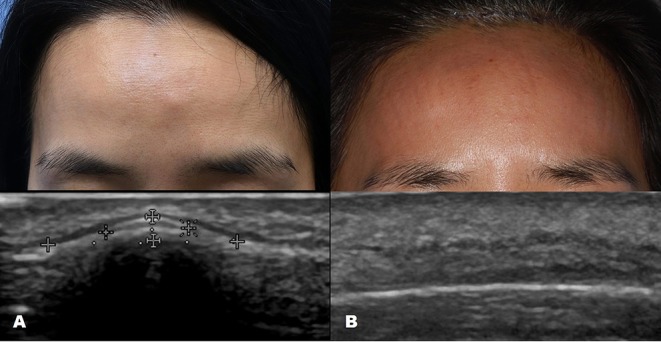
Ultrasound images of forehead osteoma. **(A)** Before excision. **(B)** After excision performed through a remote incision above the hairline.

Ultrasound imaging uses high-frequency sound waves that cannot be heard by the human ear. When it is sent inside the human body, the degree of absorption and reflection is cut off depending on the constituents and the reflected sound waves are sensed and imaged (2). Therefore, the probe that sends and detects the sound waves forms the core equipment for ultrasound technology. Higher-frequency (MHz) sound waves enable high-resolution observation of the skin surface, but the observable depth decreases. In the field of dermatology, ultrasound is mainly used to identify benign tumor type and extent (Table 3). Before surgery, it can provide information about tumor type and size, locate the existence of surrounding vessels, identify the best location for the incision, and set the range while viewing the ultrasound screen in real time with the patient. It can also help the clinician evaluate whether the tumor was completely removed after surgery (Figure 1).

**Table 3:** Key articles comparing ultrasound imaging and histopathology.

**Tumor type**	**Year**	**Main findings**	**Correlation with histopathological findings**	**Probe frequency**	**Sample size**
Basal cell carcinoma (3)	2008	1. BCC tumor ultrasound shows an oval and hypoechoic lesion 2. Compare tumor thickness measurements between ultrasound and histology	Good thickness correlation with histology (intraclass correlation coefficient, 0.9)	7–15 MHz probe	25 patients
Basal cell carcinoma (4)	2007	Lesions that may have a higher aggressive potential may also appear as hyperechoic spots	Hypersonographic spots in BCCs seemed to correspond to calcification, horn cysts, or clusters of apoptotic cells in the centers of nests of basal cell carcinoma	15 or 30 MHz	29 basal cell carcinomas
Invasive squamous cell carcinoma (5)	2009	SCC metastasized to lymph node showed asymmetrical cortical area with high elasticity	Presence of metastatic tumor cells located asymmetrically in a small section of the cortical area	Not mentioned	1 patient
Merkel cell carcinoma (6)	2017	1. Hypoechoic pattern with variable vascularization 2. Useful in the diagnostic work-up of MCC and can help more precisely delimit the tumor prior to complete surgical resection	Not mentioned	18 MHz	7 patients
Pilomatricoma (7)	2005	Well-defined mass with inner echogenic foci and a peripheral hypoechoic rim or a completely echogenic mass with strong posterior acoustic shadowing	Inner echogenic foci may relate with calcification or ossification	7–12 MHz	20 pilomatricomas from 19 patients
Trichilemmal cyst (TC) (8)	2019	Well-defined hypoechoic lesions with internal calcification and posterior sound enhancement	TC contains homogeneous eosinophilic keratinous materials Calcified foci within this keratin can be found	3–12 MHz 6–18 MHz	54 TCs from 50 patients
Ruptured epidermal cyst (REC) (9)	2008	RECs were classified into three types: with lobulations showing echogenic inner contents (type I), with protrusions (type II), and with abscess pocket formations showing poorly defined pericystic changes and increased vascularity around the abscess formation (type III)	Histopathology of the excised RECs also showed similar morphology	5–10 MHz 5–12 MHz	13 patients
Lipoma in the forehead (10)	2016	1. Hyperechoic striated septae parallel to the skin suggestive of lipoma 2. Ultrasonographic findings were accurate in 9 of 14 cases (64.3%).	Unlike the preoperative ultrasonographic findings, 13 of 14 cases were confirmed as frontalis-associated lipomas intraoperatively	12 or 15 MHz	14 patients with lipomas in the forehead

**Figure 2:**
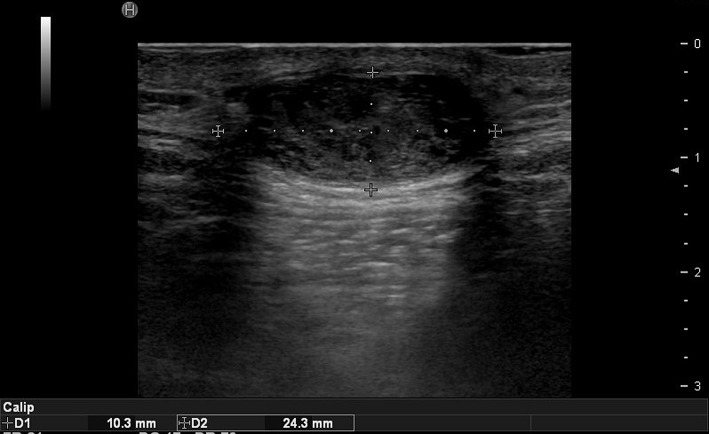
Ultrasound image of epidermal cyst.

**Figure 3:**
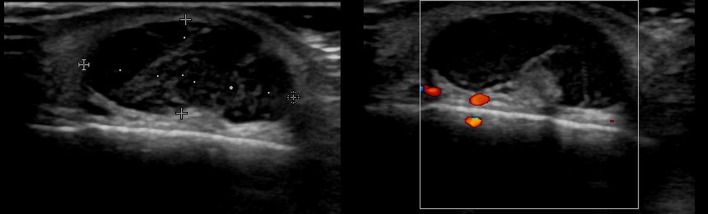
Ultrasound image of trichilemmal cyst.

In the case of epidermoid cysts, one of the most common benign tumors, it is often seen as a well-defined ovoid-shaped heterogeneous hypoechoic lesion in the subcutaneous layer with strong posterior acoustic enhancement (Figure 2). Ultrasonographic findings corresponding to epidermal cyst rupture include pericystic changes, increased vascularity, deep abscess formation, and others (9). Trichilemmal cyst, a benign appendage lesion derived from the outer root sheath of the hair follicle, is often seen as a well-defined hypoechoic lesion with internal calcification and posterior sound enhancement (Figure 3) (8). Identifying these sites just prior to surgery and optimizing the incision site and approach can improve the success rate and reduce recurrence rates.

**Figure 4:**
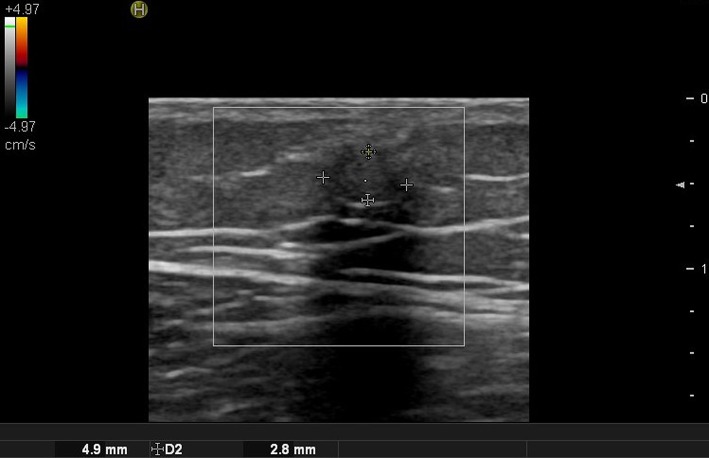
Ultrasound image of pilomatricoma.

Pilomatricoma, a benign superficial tumor of the hair follicle, is often seen as a well-defined mass with inner echogenic foci and a peripheral hypoechoic rim or a completely echogenic mass with strong posterior acoustic shadowing in the subcutaneous layer on ultrasonography (Figure 4) (7). Pilomatricoma often shows angiographic findings and may be difficult to differentiate from hemangioma.

**Figure 5:**
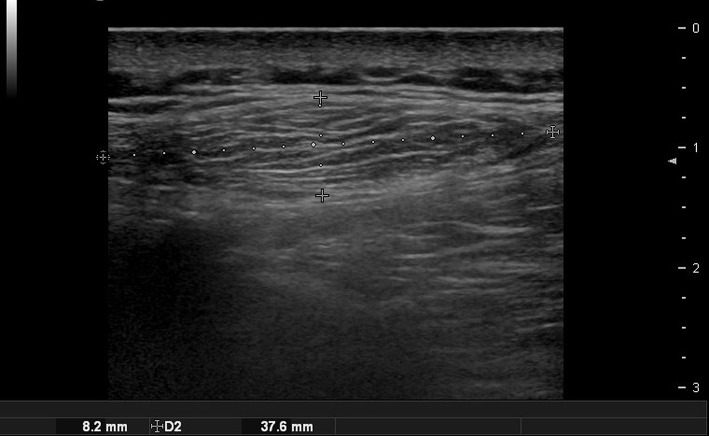
Ultrasound image of lipoma.

**Figure 6:**
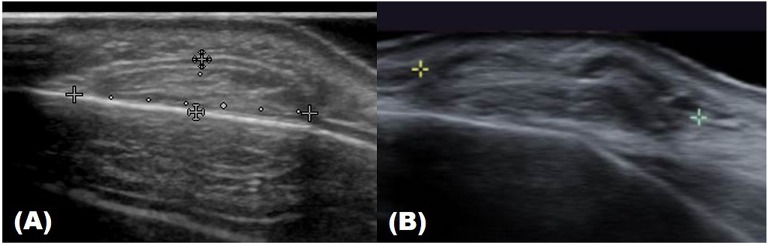
Ultrasound image of forehead lipoma. **(A)** Submuscular layer. **(B)** Subcutaneous layer.

A lipoma appears as a well-defined hypoechoic mass with multiple echogenic strands on ultrasound (Figure 5). If the encapsulation is well-formed, it is easier to remove. Ultrasonography is especially useful for diagnosing and treating lipoma in the forehead. A lipoma occurring in the forehead is often located under the frontalis muscles, and it is important to confirm its precise position using preoperative ultrasonography. It typically has a semispherical shape when located under the muscles and an ovoid shape when it is located in the subcutaneous fat layer (Figure 6) (11). However, this is not always the case, so a comprehensive judgment should be made by checking whether it is close to the periosteum or using a special technique that uses the angulation of the probe to point out the lateral borders of the lesion (12).

**Figure 7:**
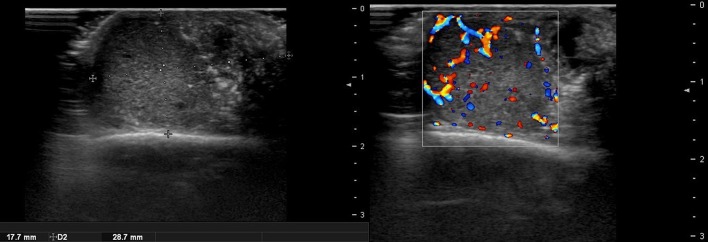
Ultrasound image of malignant proliferating trichilemmal tumor.

There are no obvious criteria that can diagnose malignant cutaneous tumors using ultrasound imaging. However, tumor size >5 cm, infiltrated margins, rapid clinical growth, moderate to severe intratumoral hypervascularity (Figure 7), and an absence of the typical features of benign tumors are highly suggestive of malignancy (13, 14). High-definition ultrasound with transducers up to 70 MHz, which can observe more detail, has been used to diagnose cutaneous angiosarcoma of the breast and is expected to be useful for the identification of malignant skin cancers (15).

## Optical Coherence Tomography

**Figure 8:**
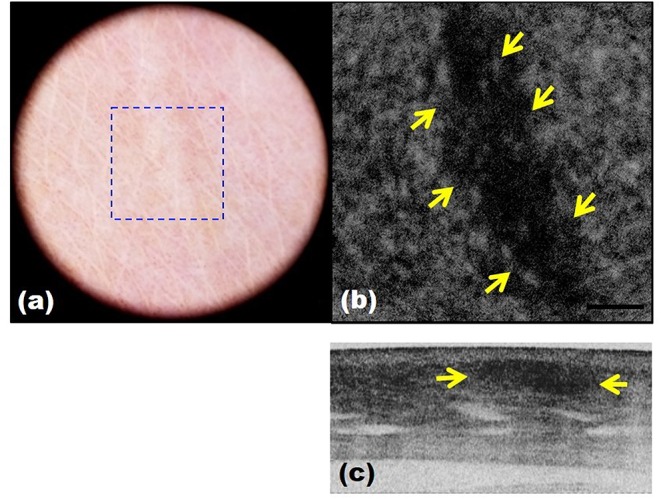
Scar images by dermoscopy-guided multifunctional optical coherence tomography (OCT). **(a)** Dermoscopic image. **(b,c)** Intensity OCT showing a dark area and frequent banding pattern due to stronger light scattering and birefringence.

**Figure 9:**
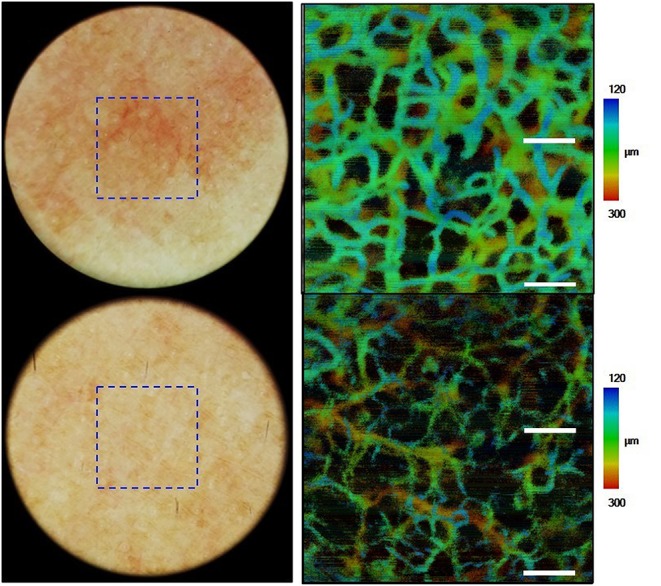
Images of nevus flammeus and normal skin acquired by dermoscopy-guided angiographic optical coherence tomography.

Optical coherence tomography (OCT), a three-dimensional (3D) imaging technique based on low coherence interferometry, creates an image by detecting the interference phenomena from light scattering or reflection as it passes through different layers of skin via the time domain or Fourier-domain method. OCT non-invasively provides skin images similar to the B mode of ultrasound to a depth of 1–2 mm and a resolution of 2–10 μm with high imaging speed. Functional OCT techniques that can provide additional information, such as polarization and vasculature were recently developed and applied for the detection of abnormal vasculature of a port-wine stain or skin cancer (16–18). Our research group developed a device that matches an OCT image with that obtained by dermoscopic imaging and provides more information than dermoscopy alone (19). Through this, we expect to be able to assess the extent of scar treatment (Figure 8). It is expected that a stage of nevus flammeus will be established, and treatment feasibility and degree will be evaluated (Figure 9). The limitations of OCT are limited depth of examination and lack of resolution to observe cancer cell morphology. Line-field confocal OCT, which can reveal comprehensive structural mapping of the skin at the cellular level with an isotropic spatial resolution of ~1 μm to a depth of ~500 μm, was recently reported to correlate with conventional histopathological images of skin tumors (20). Key articles comparing OCT and histopathology are summarized in Table 4.

**Table 4:** Key articles comparing optical coherence tomography and histopathology.

**Tumor type**	**Year**	**Type**	**Main findings**	**Correlation with histopathology findings**	**Sample size**
Basal cell carcinoma (BCC) (21)	2014	High-definition optical coherence tomography (HD-OCT)	Lobulated nodules, peripheral rimming, epidermal disarray	Peripheral rimming in HD-OCT correlates with peritumoral mucin deposition	25 cases of BCC
BCC (22)	2016	Dynamic OCT enables the detection of blood flow *in vivo* and visualization of the skin microvasculature	Blood vessels varied from dilated, larger-than normal vessels to the smallest detectable vessels	Loose and more vascularized dermis between tumor nests	1 patient with BCC on the cheek
BCC, Melanoma (20)	2018	Line-field confocal OCT	BCC: lobulated structures within the dermis, dark cleft due to mucin deposition; melanoma: general architectural disarrangement, disruption of the dermal-epidermal junction, pagetoid spread of atypical melanocytes	BCC and melanoma approximate shapes observed in OCT appeared similar histopathologically	2 patients with BCC 2 patients with melanoma
Actinic keratosis (AK), Squamous cell carcinoma (SCC) (23)	2015	HD-OCT	Absence of an outlined dermo-epidermal junction on cross-sectional images allowed discriminating SCC from AK and normal skin	It related to irregular budding of the epidermis outstanding into the upper dermis and/or presence of periadenexal collars penetrating through the dermo-epidermal junction	37 cases of AK 16 cases of SCC

## Confocal Microscopy

Confocal microscopy is based on the existence of one focal point when a laser, used as a light source, is reflected off a subject. The “out of focus” signal is blocked by a pinhole, and contrast is generated by reflections at the interfaces of tissue and cellular structures due to variations of the index of refraction. Since image acquisition is not possible with a single signal point, imaging occurs by scanning across several pinholes. Imaging up to a depth of 100–200 μm at a 1-μm resolution is possible. Confocal microscopy is capable of providing rapid bedside pathological analysis by producing images with subcellular resolution without skin biopsy and physical sectioning (24–26). There are two ways to use this approach for Mohs surgery. One is used *in vivo* and can help the identification of the surgical margins in a perioperative setting (27). It is also possible to check the remaining lesion using intraoperative images *in vivo* after removing the main skin cancer mass (28). The other is for *ex vivo* use, in which the surgical margins are removed and confocal microscopy is used to confirm whether the tumor remains within it (29). However, when used for detection in Mohs surgery, the grayscale confocal image was difficult to interpret by the surgeons. To improve this, each frozen specimen was stained with acridine orange (pH 6.0) and eosin (pH 6.0) and then scanned with confocal mosaicking microscopy to imitate hematoxylin and eosin-stained Mohs frozen sections. This approach and physician training can improve the accuracy of the non-melanoma skin cancer diagnosis (30). Key articles comparing confocal microscopy and histopathology are summarized in Table 5.

**Table 5:** Key articles comparing confocal microscopy and histopathology.

**Tumor type**	**Year**	**Type**	**Main findings**	**Correlation with histopathological findings**	**Sample size**
Basal cell carcinoma (BCC) (31)	2002	Real-time, confocal reflectance microscopy (*in vivo*)	Confocal features correlated very well with hematoxylin and eosin (H&E)-stained sections of the biopsy specimen	Features that were readily identified by both *in vivo* confocal microscopy and standard microscopy of H&E-stained sections included parakeratosis, actinic changes overlying the BCC, relative monomorphism of BCC cells, BCC nuclei exhibiting characteristic elongated or oval appearance, high nucleocytoplasmic ratios, and the presence of prominent nucleoli, increased vascularity, and prominent predominantly mononuclear inflammatory cell infiltrate	8 BCC lesions
Actinic keratosis (AK), squamous cell carcinoma (SCC), keratoacanthoma (32)	2009	Reflectance confocal microscopy (*in vivo*)	All 38 cases displayed an atypical honeycomb and/or disarranged pattern of the spinous-granular layer of the epidermis; round nucleated cells were seen in 20 SCCs (65%) and 1 AK (14%) Round blood vessels were seen in the superficial dermis in 28 SCCs (90%) and 5 AKs (72%)	Round nucleated cells at the spinous-granular layer correspond to atypical keratinocytes or dyskeratotic cells	A total of 38 lesions in 24 patients with 7 AKs, 25 SCCs *in situ*, 3 invasive SCCs, and 3 keratoacanthomas
Bowen disease (BD) (33)	2012	Reflectance confocal microscopy (*in vivo*)	Two types of targetoid cells were seen: those presenting as large, homogeneous, bright cells with a dark halo; and round ones with a dark center, surrounding bright rim, and dark halo	Targetoid cells correlated dyskeratotic cells with condensed, eosinophilic cytoplasm and a retraction halo. Dyskeratotic cells were correlated with a dark central nucleus and a surrounding clear retraction halo	10 cases of BD
BCC (34)	2013	Comparison of reflectance confocal microscopy and multiphoton tomography findings (*in vivo*)	Elongated cells and palisading structures are easily recognized using both methods	Due to the higher resolution, changes in nucleus diameter or cytoplasm could be visualized using multiphoton tomography (MPT) Therefore, nucleus diameter, nucleus/cytoplasm ratio, and cell density are estimated for normal and BCC cells using MPT	9 patients with BCC

Confocal microscopy has also been applied to diagnose mammary and extramammary Paget's disease (EMPD) (37), frequently showing Paget cells predominantly within the epidermis (38). However, due to the limited depth of imaging (100–200 μm) when applied non-invasively, the invasion site is difficult to determine. A major limitation of this technique is that it can only provide morphological information and does not reflect the tissue's internal structure or functional state.

## Two-photon Microscopy

**Figure 10:**
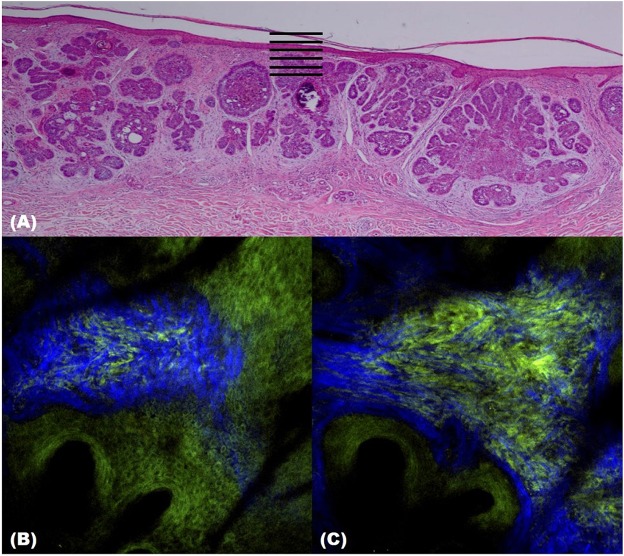
Two-photon microscopy (TPM) images of basal cell carcinoma (BCC). **(A)** Histopathological finding. **(B,C)** TPM images showing parallel collagen fibers (blue) surrounding a BCC tumor nest.

Two-photon microscopy (TPM) is a technique that uses the fluorescence released after excitation from simultaneously absorbing two photons with long wavelengths and low energy. TPM allows observation of vital phenomena in cells and *in vivo* at the molecular level. In particular, it has the advantage of being able to identify the distribution of collagen within the dermis using the second harmonic generation (SHG) produced when two photons simultaneously interfere. Non-invasive *in vivo* multi-photon microscopy (MPM) imaging also reportedly provides label-free contrast and reveals several characteristic features of basal cell carcinoma lesions (39). This feature correlates well with histopathological examination, findings, and SHG in particular shows collagen and elastin bundles around the tumor (Figure 10) (Table 6).

**Table 6:** Key articles comparing multiphoton microscopy and histopathology.

**Tumor type**	**Year**	**Type**	**Main findings**	**Correlation with histopathological findings**	**Sample size**
Basal cell carcinoma (BCC) (39)	2015	*In vivo* multiphoton microscopy (MPM)	1. Nests of basaloid cells palisading in the peripheral cell layer at the dermoepidermal junction and/or in the dermis 2. Parallel collagen and elastin bundles surrounding the tumors 3. Mucinous stroma adjacent to tumor was visualized using MPM	These features generally correlated well with histopathologic examination. However, histologic examination revealed palisading of peripheral layers in some of the tumor nests of the lesion, although this feature was not obvious in the nests imaged with MPM.	9 patients with a total of 10 BCC
Squamous cell carcinoma *in situ* (SCCIS), superfical BCC (SBCC) (40)	2008	*Ex vivo* MPM	The following findings were seen: SCCIS: bowenoid dysplasia, multinucleated cells, or hyperkeratosis SBCC: peripheral palisading of tumor cells	The morphologic features differed significantly between these lesions and perilesional skin.	5 specimens of SCCIS 6 specimens of SBCC
Actinic keratosis (AK), squamous cell carcinoma (SCC) (41)	2016	*In vivo* MPM	Changes in the morphology of the keratinocytes, such as broadened epidermis, large intercellular spaces, enlarged nucleus and a large variance in cell shape could easily be recognized.	AK: hyperparakeratosis and cell pleomorphism SCC: invasion of the dermis, keratin pearls and hyperchromatic nuclei	6 patients with AK 6 patients with SCC
Benign and malignant melanocytic nevi (BMMN) (42)	2014	*In vivo* MPM	They evaluate BMMN using 9-point scale showing different values according to two-photon excited fluorescence and second harmonic generation of nevi. Indices corresponding to common nevi (0–1), dysplastic nevi (1-4), and melanoma (5-8) were significantly different (*P* < 0.05).	Prominent qualitative correlations included the morphology of epidermal keratinocytes, the appearance of nests of nevus cells surrounded by collagen fibers, and the structure of the epidermal–dermal junction.	5 common nevi 5 dysplastic nevi 5 melanoma
BCC, SCC, dermatofibrosarcoma protuberans (DFSP) (43)	2019	*Ex vivo* moxifloxacin labeling-based MPM	Moxifloxacin MPM imaged both cells and collagen in the skin, similarly to label-free MPM, but with enhanced fluorescence intensities in cells and enhanced imaging speeds.	Moxifloxacin MPM could detect specific cellular features of various skin cancers in good correlation with histopathological images at the higher imaging speed than label-free MPM.	10 patients with BCC 1 patient with SCC 1 patient with DFSP

**Figure 11:**
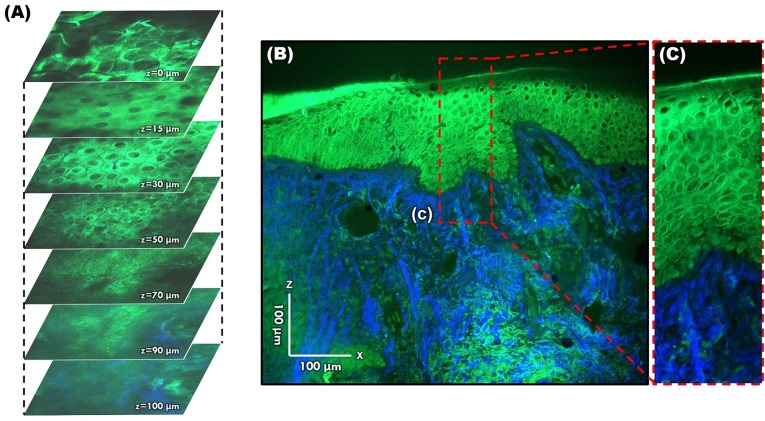
Moxifloxacin-based multi-photon microscopy images of normal skin. **(A)** En face images at different depths. **(B,C)** Cross-sectional view of the epidermis and dermis.

However, since TPM and MPM utilize weak endogenous fluorescence in tissue, there is a need for high excitation laser power and extension of pixel duration (44, 45). To overcome this limitation and reduce photodamage, moxifloxacin, an FDA-approved antibiotic, has been reported as a cell-labeling agent for MPM (46). Moxifloxacin has bright intrinsic multi-photon fluorescence, good tissue penetration, and high intracellular concentration. In addition, moxifloxacin-based MPM imaging is 10 times faster than imaging based on endogenous fluorescence (Figure 11) (46).

Although imaging depth remains a limitation, various methods to achieve a clear and high-resolution image are being developed. It is also expected that the diagnosis rate can be increased by tumor marker labeling. A recent report stated that in patients with EMPD, a subclinical extension can be assessed by MPM using whole-mount immunostaining with anti-cytokeratin 7 antibody to label Paget cells (35). These trials will be used in the *ex vivo* skin tissue to find the tumor's margins, and it is anticipated that it may replace frozen sections in the future. For more generalized clinical applications, the cost of the equipment is the greatest hinderance. MPM equipment is expensive because it uses a femtosecond laser (36).

## Conclusion

In addition to ultrasonic devices that can closely observe the skin and deep structures, the development of dermatological equipment that unites laser and optical technology has shown visible progress. The principle of these devices is to analyze signals reflected or scattered from the skin, and there is a fundamental limitation that it is evaluated by looking into the mirror. These limitations are expected to improve in the near future by the development of fluorescent probes targeting tumors or diseases and will be used more actively for the diagnosis and treatment of skin lesions.

For dermatologists, this is a good opportunity to strengthen the specialty of dermatology. We are already familiar with laser equipment and have demonstrated a correlation between clinical and histopathological findings. When we use imaging equipment to further investigate a patient's skin and present objectively explainable data by linking “clinical imaging–histopathological findings,” a more robust doctor–patient relationship can be established.

## Author Contributions

BO conceived the concept and wrote the manuscript. KK co-conceived the concept and drafted the figures and tables. KC co-conceived the concept and edited and improved the manuscript.

### Conflict of Interest

The authors declare that the research was conducted in the absence of any commercial or financial relationships that could be construed as a potential conflict of interest.

## Footnotes

^1^Available online at: http://obel.ee.uwa.edu.au/research/fundamentals/introduction-oct/

## Abbreviations

OCT: optical coherence tomography
TPM: two-photon microscopy
EMPD: extramammary Paget's disease
SHG: second harmonic generation
MPM: multi-photon microscopy
BCC: basal cell carcinoma
SCC: squamous cell carcinoma
AK: actinic keratosis.
